# Critical Product Features' Identification Using an Opinion Analyzer

**DOI:** 10.1155/2014/340583

**Published:** 2014-11-24

**Authors:** Azra Shamim, Vimala Balakrishnan, Muhammad Tahir, Muhammad Shiraz

**Affiliations:** ^1^Faculty of Computer Science and Information Technology, University of Malaya, Kuala Lumpur 50603, Malaysia; ^2^COMSATS Institute of Information Technology, Islamabad 44000, Pakistan; ^3^Federal Urdu University of Arts, Science and Technology, Islamabad 44000, Pakistan

## Abstract

The increasing use and ubiquity of the Internet facilitate dissemination of word-of-mouth through blogs, online forums, newsgroups, and consumer's reviews. Online consumer's reviews present tremendous opportunities and challenges for consumers and marketers. One of the challenges is to develop interactive marketing practices for making connections with target consumers that capitalize consumer-to-consumer communications for generating product adoption. Opinion mining is employed in marketing to help consumers and enterprises in the analysis of online consumers' reviews by highlighting the strengths and weaknesses of the products. This paper describes an opinion mining system based on novel review and feature ranking methods to empower consumers and enterprises for identifying critical product features from enormous consumers' reviews. Consumers and business analysts are the main target group for the proposed system who want to explore consumers' feedback for determining purchase decisions and enterprise strategies. We evaluate the proposed system on real dataset. Results show that integration of review and feature-ranking methods improves the decision making processes significantly.

## 1. Introduction

With the overwhelming popularity of Web2.0, electronic word-of-mouth transforms into a new, mostly free-of-charge opinion data wherein users present opinions and experiences to a wide public. This revolution has converted passive information users into actors as they create the content of the web themselves. As a result, consumers' interconnections through blogs, online forums, newsgroups, and consumers' reviews become a global phenomenon that facilitates the dissemination of both positive and negative word-of-mouth [1]. Existing literature shows that positive word-of-mouth helps to improve consumers' satisfaction, trust, and loyalty [2]. However, negative word-of-mouth reduces consumers' loyalty and patronage [3]. Electronic word-of-mouth resulted in a generation of sophisticated and knowledgeable shoppers [4]. Therefore, consumers and enterprises acquire online reviews to adapt purchase intentions and business models and reorganize and prepare future plans [5].

Consistently and rapidly growing word-of-mouth (online reviews) presents tremendous opportunities and challenges for consumers and marketers. It empowers consumers to carry out more informed decisions about purchases. However, it challenges marketers to create interactive marketing campaigns that can effectively connect with and engage target consumers [4]. Viral marketing is the most intriguing strategy among consumer-leveraging possibilities that the Internet offers [6]. The goal of viral marketing is to utilize consumer-to-consumer communications for disseminating information about a product or service. One potential tool for viral marketing is opinion mining system, which helps consumers and enterprises to identify the strengths and weaknesses of products using consumer-to-consumer communications. It supports diverse businesses-intelligence tasks including sales prediction, reputation management, threats analysis from competitors and enterprise risks, support decision making and risk management, design new products, and marketing strategies.

Online reviews vary greatly in quality, resulting in information overload problem [7–9]. Consequently, it becomes difficult to identify high quality reviews. For this purpose, some review websites such as http://www.amazon.com/ ask users to rate review and votes for their helpfulness. Users explicitly filter reviews based on rating (ranges from 1 to 5 stars) and/or helpfulness votes (how many people found the review helpful) in order to obtain high quality informative reviews [10, 11]. The quality of a review is a property orthogonal to its polarity or embedded opinions [12] or how helpful a review is [13]. However, most of the existing opinion mining systems ignore the quality of reviews and disregard opinion strength that defines how positive or negative an opinion is [2]. A detailed feature-level summary of a product is required for satisfying consumers and enterprise demands wherein prominent features are ranked according to the consumers' opinions.

This paper is devoted to integrating review and feature ranking methods for providing consumers and enterprises with high quality decision-oriented information from enormous consumers' reviews. The objectives of this paper are to (i) select high quality informative reviews using metadata and semantic features according to users' preferences and (ii) propose a new approach for feature ranking based on the intensity of opinion in order to identify critical product features.

The rest of the paper is organized as follows.  Section 2 discusses existing work on review quality evaluation and feature ranking.  Section 3 presents the proposed system.  Section 4 includes results and discussion. The conclusion of the paper is presented in Section 5.

## 2. Related Work

This section presents existing work on review quality evaluation and feature ranking techniques.

### 2.1. Review Quality Evaluation

The high volume of reviews makes it harder for consumers and businesspersons to locate best reviews and analyze the true underlying quality of a product. Currently, different studies are conducted to predict review quality according to various features such as helpfulness votes, rating, length, and term frequency (how often a term appeared in a review). In [13], the authors predicted the quality of a review according to helpfulness votes based on five feature classes: structural (length and number of sentences), lexical (unigram and bigram), syntactic (nouns, adjectives, and verbs), semantic (features and opinion words), and metadata (rating) on the helpfulness of a review. The experimental results show that review length, users' rating, and term frequency are significant in review quality prediction. The shallow syntactic features (nouns, verbs, and interjections) were found to have the strongest impact on review quality prediction in [12]. Another study is conducted to investigate the impact of three additional sets of features including reviewer profile features, reviewer history features, and readability features on review helpfulness [14]. Results show that these features affect the perceived helpfulness of reviews. Liu et al. showed that the helpfulness of a review depends on reviewer's expertise, writing style of the review, and timeliness of the review [15]. The number of product features and review length are also found significant for review ranking [7]. In [16], the authors ranked book reviews according to the number of features. A score is assigned to each review based on the number of features discussed in the review and the reviews are ranked based on the score. The proposed method outperformed the conventional helpfulness votes based ranking method. The number of features and opinion words are utilized in [17] for review ranking. The results show improvement over term frequency based method.

### 2.2. Feature Ranking

Feature ranking highlights the strengths and weaknesses of a given product to support consumers in their purchase decisions. It also assists entrepreneurs in business-intelligence tasks such as sales prediction, reputation management and risk management, and developing new products and marketing strategies. Existing feature ranking approaches are based on feature frequency, the number of associated opinion words, rating, and the number of positive and negative comments [17–20]. The feature frequency is utilized in [18] to rank features, which results in better performance than the method proposed in [21]. Similarly, the PageRank algorithm is adapted in [22] for product ranking based on feature frequency. Experimental results show that the proposed method achieved significant agreement with experts' evaluations. Eirinaki et al. [17] ranked features from a different perspective wherein a score is assigned to each feature based on the number of associated opinion words. The proposed method outperformed the frequency based method. Similarly, features are ranked according to associated opinion words and the ranking guidelines provided by the review website [23], which improves the accuracy of the feature-based summarization system [24]. In [19], the authors proposed the use of rating with opinion words to rank product features and results demonstrate the superiority of the proposed method over frequency based method [25]. Similarly, rating and the number of positive and negative comments for feature ranking are found to produce higher precision than frequency based method [25]. The number of positive and negative comments with feature frequency is deployed to rank feature in [20] which achieves 92% precision.

## 3. Research Methodology

**Figure 1:**
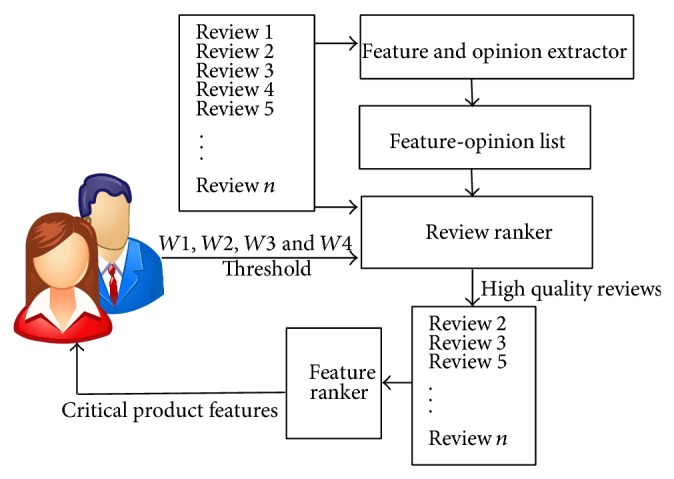
Architecture of the opinion analyzer.

This section describes the architecture of the proposed system called opinion analyzer. The main components of the opinion analyzer are shown in Figure 1. It consists of three components: (i) feature and opinion extractor, (ii) review ranker and (iii) feature ranker.

In the first step, product features and the opinion words that are used to describe such features are extracted from reviews and are stored in a file. Reviews are ranked according to rating, helpfulness vote, and the number of features and opinion words according to user preferences. The reviews having less rank than a threshold value are discarded and the remaining high quality reviews are used for feature ranking. Finally, positive and negative ranks of a feature are calculated using opinion intensity from the high quality reviews.

The purpose of the feature and opinion extractor is to extract product feature and associated opinion words. Part of speech tagging (POS) is performed to generate feature list. Then, a file which contains the most frequent identified nouns and noun phrases is built based on the idea that product features are usually frequent nouns or noun phrases [16, 20, 23]. To extract associated opinion words of the frequent features, we used a window of size *K*, which means opinion words that are within *K* words of the selected feature are considered as associated opinion words, based on the assumption that an opinion word associated with a feature is mentioned in its vicinity [25]. After the extraction of opinion words, a file is maintained which contains identified feature and opinion words.

The review ranker then assigns weights to the reviews using (1), where FC is the number of features in the review, OWC is the number of opinion words in the review, rating is the rating, and HR is the helpfulness ratio of a review. HR indicates the percentage of people who find the review helpful. Users can define the contribution of each parameter by the values of  *W*1, *W*2, *W*3, and *W*4, in order to incorporate their preferences. In (1), all weights are summed to 1. Consider

(1) Rweight=W1∗FC+W2∗OWC+W3∗rating +W4∗HR.

The review document may contain reviews having no significant impact on feature ranking [2]; therefore, detecting and filtering low-quality reviews can improve feature ranking [7]. Therefore, inspired from the review quality categories of [2, 7], current study classified the reviews into five review quality classes: excellent, good, average, fair, and poor. Informative reviews contain a number of opinions on the features, which are found more helpful. They also have a higher rating. High quality reviews (excellent and good reviews) present in-depth opinions on product features in order to make them productive for opinion summarization. The medium-quality reviews (average and fair reviews) provide few opinions on products or features whilst the low-quality reviews can contain little information about a product/feature, or the information is too objective. It is highlighted that top 5 features account for 80% to 100% of high quality reviews; therefore, low-quality reviews can be excluded from opinion summary [2, 7]. The filtration of low-quality reviews from the review document depends on the users' preferences that define review class(es) utilized by the system for feature ranking. For instance, if a user selects excellent and good reviews then the feature ranking is applied only on these particular review classes.

The feature ranker calculates two ranks for every feature including positive and negative ranks using opinion intensity. Every opinion word that appears in the selected reviews is assigned an empirical value manually (opinion intensity) that determines how positive or negative an opinion word is based on the classification provided in [26, 27]. There are six classes: weakly positive (WP), mildly positive (MP), strongly positive (SP), weakly negative (WN), mildly negative (MN), and strongly negative (SN). The opinion intensity ranges between +1(WP) and −3(SN). For instance, +3 is assigned to “*excellent*” and +1 is assigned to “*good*”. Similarly, −3 is assigned to “*terrible*” and −1 is assigned to “*bad*”.

The positive rank (*P*_rank⁡_) of a feature reflects the accumulated strength of the associated positive opinion words. It is calculated using (2) as shown below:

(2) $$\begin{array}{c} P_{\mathrm{rank}}=\overset{m}{\underset{i=1}{\sum }}{\text{OWP}}_{i}+2\overset{n}{\underset{j=1}{\sum }}{\text{OMP}}_{j}+3\overset{o}{\underset{k=1}{\sum }}{\text{OSP}}_{k}. \end{array}$$

In (2), OWP_*i*_ is the occurrence of every weakly positive opinion word, OMP_*j*_ is the occurrence of every mildly positive opinion word, and OSP_*k*_ is the occurrence of every strongly positive opinion word. Larger value of *P*_rank⁡_ indicates that the selected feature is discussed more positively by the users.

The negative rank (*N*_rank⁡_) of a feature encodes the accumulated strength of associated negative opinion words. The *N*_rank⁡_ of a feature is calculated using

(3) $$\begin{array}{c} N_{\mathrm{rank}}=\overset{m}{\underset{i=1}{\sum }}{\text{OWN}}_{i}+2\overset{n}{\underset{j=1}{\sum }}{\text{OMN}}_{j}+3\overset{o}{\underset{k=1}{\sum }}{\text{OSN}}_{k} \end{array}$$

whereas OWN_*i*_, OMN_*j*_, and OSN_*k*_ are the occurrences of every weakly negative, mildly negative, and strongly negative opinion words, respectively. The intuition behind the *N*_rank⁡_ is that if a feature is described by more negative words then it should be ranked higher than others. Larger value of *N*_rank⁡_ indicates that the users discuss the feature more negatively.

**Figure 2:**
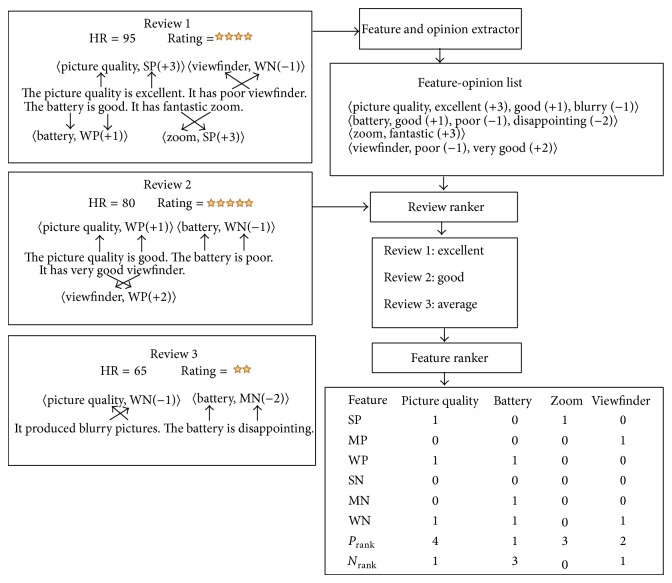
An illustrated example of the review and feature ranking.

Figure 2 shows working of the proposed methods wherein a digital camera is regarded as an object. Three reviews are considered in the example. In the feature and opinion extraction step, the features and associated opinion words are extracted and a feature-opinion list containing the extracted information is created. This list shows the extracted features: picture quality, battery, zoom, and viewfinder along with the associated opinion words with their corresponding opinion intensity. In these reviews, the feature “*picture quality*” is described by two positive opinion words (excellent and good) and one negative opinion word (blurry). The opinion words “*excellent,*” “*good,*” and “*blurry*” are strongly positive (+3), weakly positive (+1), and mildly negative (−2), respectively; hence, the SP, WP, and MN counts are set to one. One positive opinion word “*good*” and two negative opinion words “*poor*” and “*disappointing*” are associated with the feature “*battery.*” The opinion strength of the opinion words is weakly positive (+1), weakly negative (−1), and mildly negative (−2), respectively; hence, the WP, WN, and MN counters are set to one. The feature “*zoom*” is defined by the opinion word “*fantastic*,” which is a strongly positive opinion word with an opinion strength of +3; as a result the value of one is assigned to the SP counter of the feature. One weakly negative opinion word “*poor*” and one mildly positive opinion word “*very good*” are related to the feature “*view finder*” and subsequently, the WN and MP counters are set to one. Finally, the values of SP, MP, WP, SN, MN, and WN are utilized to compute *P*_rank⁡_ and *N*_rank⁡_ of each feature using (2) and (3) as shown in Figure 2. Secondly, the review quality class of each review is calculated using (1) and is shown in Table 1, assuming equal weight (0.25) for all parameters.

The accuracy of the system is calculated by comparing the values of *N*_rank⁡_ and *P*_rank⁡_ generated by the system with the manually calculated values to check the effectiveness of the system. For instance, in Figure 2, the manually calculated *P*_rank⁡_ of picture quality is 4; and if the system calculated value is 3, the accuracy is given by (3/4)∗100.

## 4. Experimental Data

The opinion analyzer based on the proposed review and feature ranking methods is implemented in Python, which is a programming language used for natural language processing. The input to the system is a text file, which contains reviews. The opinion analyzer extracts helpfulness votes, rating, features, and opinion words from reviews and ranks reviews using (1) discussed in Section 3. Low-quality reviews are discarded and the remaining high quality reviews are used for feature ranking in which features are ranked according to the number of positive and negative comments using opinion intensity. The output of the opinion analyzer is prominent feature of a given product along with positive and negative ranks and review quality classification of input reviews.

Reviews from http://www.amazon.com/ are utilized for experiments in this work.  Box 1 shows a sample review of Canon G3 PowerShot camera from http://www.amazon.com/ that consists of helpfulness votes, users' rating, reviewer name, title, and body of review. We have conducted experiments on data file of Canon G3 camera [23, 26, 27], which consists of 45 reviews from http://www.amazon.com/ for the evaluation of opinion analyzer. In the file, the tag [*t*] (inserted by [23]) indicates that the sentence following [*t*] is the title of the review (Box 2). Further, a new line in the review is expressed with “##” symbol. Features are marked with their corresponding opinion strength and opinion orientation in the data file; for instance, the feature “*picture*” is marked with a positive opinion intensity of +2 as the bold font in Box 2. However, both the helpfulness ratio and rating are not available in the data set (Box 2) used by [23, 26, 27]. Therefore, these are calculated and added in the current study.

The helpfulness ratio (helpfulness votes/total votes ∗ 100) of a review is calculated from the helpfulness votes of a review (provided by http://www.amazon.com/) for each review in the file; for instance, the helpfulness ratio of the review shown in Box 1 is 75 (3/4∗100) as three people out of four found the review helpful. Two tags, namely [*h*] and [*r*], are introduced to represent helpfulness ratio and users' rating, respectively.  Box 3 shows the final review after the helpfulness ratio and ratings are included, in which [*h*][75] represents the helpfulness ratio (75%) and [*r*][5] describes the rating of the review, that is, 5 stars.

## 5. Results and Discussion

This section presents review quality classification of Canon G3 reviews, top ten features of Canon G3 camera according to positive and negative comments based on the methods described in Section 3.

**Figure 3:**
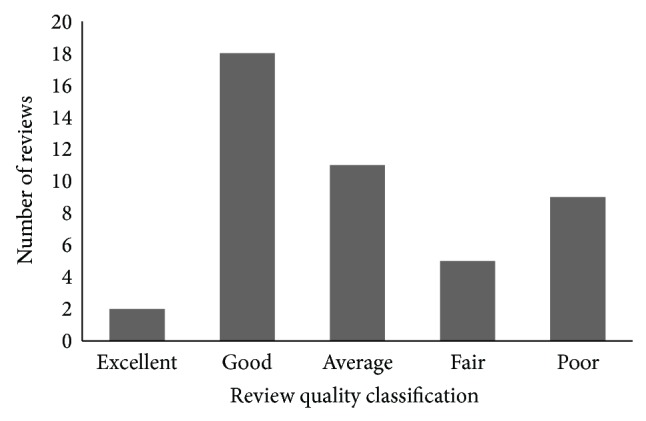
Review quality classification of Canon G3.

Figure 3 shows the review quality classification of  “Canon PowerShot G3” data file. The file consists of mix quality reviews; however, most of the reviews belong to “*good*” review quality class. Short reviews with less features and opinions having fewer helpfulness votes and rating result in low review quality. Furthermore, a majority of the reviews (64%) are “*good*” and “*average*” reviews that provide adequate amount of information about features and consumers' opinions. Only 31% reviews are categorized as “*fair*” and “*poor.*” After classifying the reviews according to their quality, users can discard low-quality reviews and apply feature ranking only on the selected review quality class(es); for instance, a user can select excellent and good reviews for feature ranking only.

**Figure 4:**
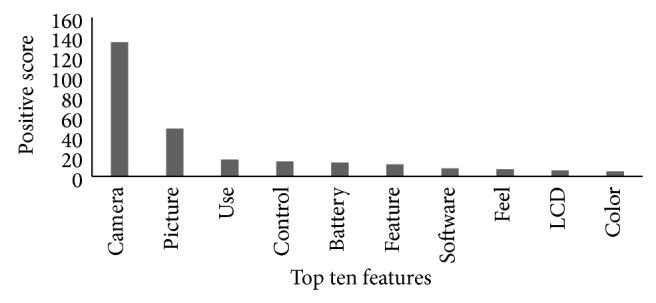
Top 10 features according to *P*_rank⁡_.

Figure 4 presents the top ten features of the “Canon G3 PowerShot” Camera according to the positive rank, which highlights the strengths of the camera. The top three features are camera, picture, and use. Overall the camera received remarkable positive feedback (around 150 positive score) than the other top ten positive features, which indicates that a great deal of consumers is satisfied with the camera. Reference [25] reported camera as a second prominent keyword for digital camera domain. The feature “picture” is at second position in the ranking, which reflects that consumers appreciated the picture quality of the camera to a large extent [25]. The feature “picture” is also in the top ten features defined by [25]. Similarly, picture is at the first position in the positive ranking described in [20]. According to http://www.ebay.com/ picture is a significant feature and it has an immense impact on future sale. The next feature is “use,” which encodes the ease of use of the camera. Consumers also favourably supported the use of the camera. The ease of use is positively related to customers' product adoption and sale generation of a product [28]. Other features such as control, battery, feature, and software are also acknowledged by consumers. In [25], battery was listed among the top features of a digital camera. Similarly, PC mag described the controls of a camera to be significant for other customers' purchase decision. “Feel,” “LCD” (liquid crystal display), and “picture colour” features are at the lowest rank in the top ten features. LCD is found to be a decisive feature in digital camera domain [20]. Similarly, LCD is declared one of the prominent features of a digital camera by PC mag that influences consumers purchase intentions. One of the popular review website, http://www.ebay.com/, analysed consumers' reviews (http://www.ebay.com/) and presents the rating of vital features, such as picture quality and features of a camera. The highlighted features have a positive impact on the sale of the camera. These top ten features are emphasized by manufacturers in marketing campaigns for the promotion of the camera. PC mag described that LCD has a significant effect on consumers purchase intentions.

**Figure 5:**
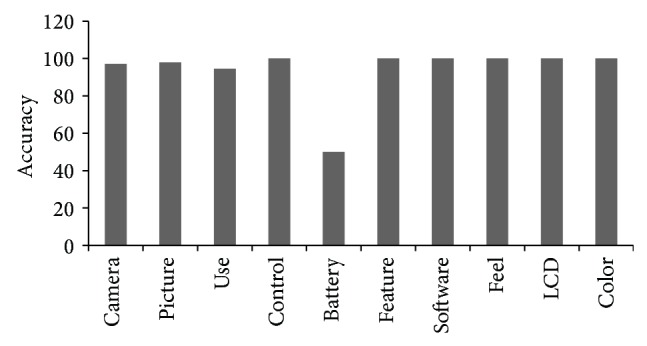
Accuracy of top ten features according to *P*_rank⁡_.

We compare the results of *P*_rank⁡_ generated by the Opinion Analyzer with manual calculation of *P*_rank⁡_ for assessing the effectiveness of the system. The accuracy of top ten features according to *P*_rank⁡_ is found 94%, which is a very good performance of the system (Figure 5).

**Figure 6:**
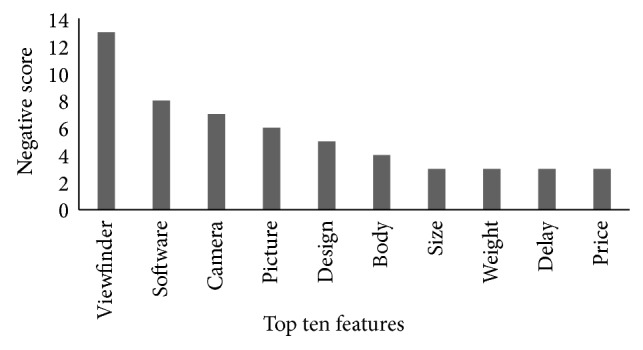
Top 10 features according to *N*_rank⁡_.

Figure 6 highlights the top 10 features of the “Canon G3 PowerShot” Camera according to the negative rank to pinpoint the weaknesses of the camera. The “viewfinder” feature is at the top after receiving more negative comments than the other to ten features, which reflects consumers' disapproval. PC mag highlighted a positive correlation of viewfinder on consumers buying behaviour. The second feature in the negative ranking with which consumers' are disagreed is software. The next feature is camera that received some negative comments and becomes third in the negative ranking. Similarly, some of the consumers are dissatisfied with pictures of the camera in addition to positive evaluation. Consumers also dissatisfied with the design and body of the camera. Other features such as size, weight, delay, and price are ranked lowest with the same negative score. Ahmad and Doja narrated price as a crucial feature [20]. According to http://www.ebay.com/, price and weight are critical for product adoption. These features are considered by manufacturers for the improvement of the camera. Another review website http://www.bhphotovideo.com/ provides the description of a digital camera in terms of battery and shutter delay. According to the guidelines provided by http://www.which.co.uk/ size, weight, and shutter delay need to be considered while making a purchase decision.

From Figures 4 and 6, it is concluded that most features are discussed positively by consumers. The features of the Canon G3 received more positive comments as compared to negative ones. Some of the prominent features, for instance, camera, picture, and software, appear in the top ten features according to positive and negative ranking and thus show a mixed assessment by consumers.

**Figure 7:**
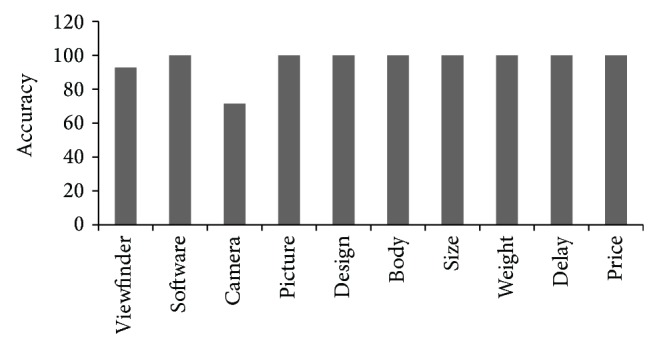
Accuracy of top ten features according to *N*_rank⁡_.

The accuracy of top ten features according to *N*_rank⁡_ is calculated by comparing the results generated by the system with the manual calculation, resulting in 96% of overall accuracy.  Figure 7 describes the accuracy of top ten features according to *N*_rank⁡_.

## 6. Contributions

The key contributions of this work are as follows.

- Our review ranking method is based on a superset of the elements used for review ranking and the elements account for most review helpfulness prediction power, that is, the integration of rating and helpfulness votes with the number of features and opinion words. This integration facilitates identification of the most important reviews from a manufacturer's point of view for corporate decision making.
- Second contribution is the involvement of stakeholders in review ranking process for defining the influence of every factor in review ranking.
- Thirdly, only high quality reviews above a threshold value defined by users are considered for the feature ranking task.
- The consideration of opinion strength in the feature ranking task is our fourth contribution.

## 7. Implication for Entrepreneurs and Consumers

The primary purpose of this work is to apply opinion mining techniques to analyze critical product features, which have significant impact on future sales, reputation management, decision making and risk management, new products' design, marketing strategies, and product adoption. Our proposed system can have several implications for entrepreneurs and consumers. The first implication of the proposed system from a business perspective is that entrepreneurs can identify critical product features that have a significant impact on sales. Secondly, this study empowers businesses to be more aware of the consensus surrounding their business and can formulate actions to resolve negative word-of-mouth and swing consumers' opinions in their favor. Thirdly, the proposed system provides an opportunity for business in the analysis of customer opinions' to identify analytical product features that are highly influential in business-intelligence tasks such as sales prediction, reputation management, and future plans. The fourth implication is for entrepreneurs who seek to venture consumer-to-consumer communications for advertisement and marketing campaigns, for instance, viral marketing, to create awareness, trigger interest, and generate sales or product adoption. Additionally, from a consumer point of view, it highlights the strengths and weaknesses of a product for making purchase decisions.

## 8. Conclusion, Limitation, and Future Work

The ubiquity of Web2.0 with the proliferation of blogs and social networks transforms the way people express their opinions about different entities, which facilitates the dissemination of word-of-mouth. Electronic word-of-mouth has become a powerful source of information for consumers and businesses that gauge consumers' purchase intentions and enterprises' strategies. Existing literature indicates that viral marketing is the most intriguing marketing strategy, which utilizes consumer-to-consumer communications to disseminate information about a product or service. One potential tool for viral marketing is the opinion mining system, which highlights the strengths and weaknesses of products using consumers' reviews. Online reviews have grown at a fast pace and vary greatly in quality; consequently, it has become imperative to identify high quality reviews to enhance the decision making process. This research work proposed review and feature ranking methods based on user preferences and opinion intensity, respectively, in order to highlight prominent product features on which users expressed their feedback from high quality reviews. The proposed methods are evaluated on a set of Amazon reviews of a digital camera. Camera, picture, and use are the top three features of the camera according to positive comments, while viewfinder, software, and camera are the top three features of the camera according to the negative comments. The proposed methods highlight the strengths and weakness of the digital camera, which assist consumers in their purchase decision and entrepreneurs in their business decision making process.

Some final remarks are concerned with potential limitations of the study. The study only analyzed electronic product reviews. The proposed system can be applied to other domains including book reviews and hotel reviews. The obvious limitation of the work is the use of http://www.amazon.com/ reviews. The proposed methods can be generalized to analyze reviews from other websites such as http://www.epinions.com/?sb=1. In this work, we tested our system with 332 reviews of five electronic products. Future replication of this work might consider the generalization of the system for other reviews formats. Future research should also analyze a large number of reviews from different domains.

## Figures and Tables

**Box 1:**
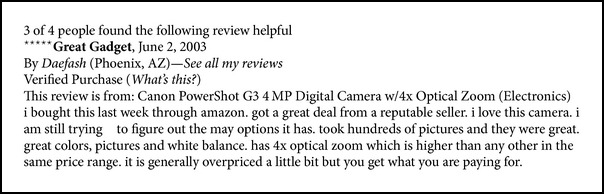
**Box 1: **A sample review of Canon G3 camera from http://www.Amazon.com/.

**Box 2:**
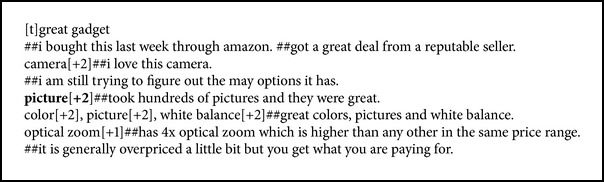
**Box 2: **A sample review of Canon G3 camera from the data file.

**Box 3:**
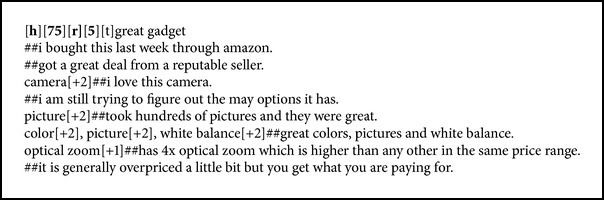
**Box 3: **Review after adding helpfulness ratio and rating.

**Table 1:** Reviews quality classification.

Reviews	Number of features (FC)	Number of opinion words (OWC)	Helpfulness ratio	Rating	Review quality
Review 1	4	4	95	4	Excellent
Review 2	3	3	80	5	Good
Review 3	2	2	65	2	Average
